# System Biology Approach to Identify the Hub Genes and Pathways Associated with Human H5N1 Infection

**DOI:** 10.3390/vaccines11071269

**Published:** 2023-07-21

**Authors:** Raushan Kumar Chaudhary, Ananthesh L., Prakash Patil, Uday Venkat Mateti, Sanjit Sah, Aroop Mohanty, Rama S. Rath, Bijaya Kumar Padhi, Sumira Malik, Kadhim Hussein Jassim, Moustafa A. Al-Shammari, Yasir Waheed, Prakasini Satapathy, Joshuan J. Barboza, Alfonso J. Rodriguez-Morales, Ranjit Sah

**Affiliations:** 1 Department of Pharmacy Practice, NGSM Institute of Pharmaceutical Sciences (NGSMIPS), Nitte (Deemed to be University), Mangaluru 575018, Karnataka, India; chaudharyrosen91@gmail.com (R.K.C.); anantheshl22@gmail.com (A.L.); 2 Central Research Laboratory, K.S. Hegde Medical Academy (KSHEMA), Nitte (Deemed to be University), Mangaluru 575018, Karnataka, India; prakashpatil@nitte.edu.in; 3 Global Consortium for Public Health and Research, Datta Meghe Institute of Higher Education and Research, Jawaharlal Nehru Medical College, Wardha 442001, India; sanjitsah101@gmail.com; 4 Department of Clinical Microbiology, All India Institute of Medical Sciences, Gorakhpur 273008, India; aroopmohanty7785@yahoo.com; 5 Department of Community Medicine and Family Medicine, All India Institute of Medical Sciences, Gorakhpur 273008, India; amashankar.aiims@gmail.com; 6 Department of Community Medicine and School of Public Health, Postgraduate Institute of Medical Education and Research, Chandigarh 160012, India; bkpadhi@gmail.com; 7 Amity Institute of Biotechnology, Amity University Jharkhand, Ranchi 834001, Jharkhand, India; smalik@rnc.amity.edu; 8 School of Applied and Life Sciences, Dehradun 248007, Uttarakhand, India; 9 Guru Nanak College of Pharmaceutical Sciences, Chakrata Road, Dehradun 248007, Uttarakhand, India; 10 Al-Mustaqbal University College, Hillah 51001, Babylon, Iraq; kadhim.hussien@uomus.edu.iq (K.H.J.); moustafa.ali@uomus.edu.iq (M.A.A.-S.); 11 Office of Research, Innovation, and Commercialization (ORIC), Shaheed Zulfiqar Ali Bhutto Medical University, Islamabad 44000, Pakistan; 12 Gilbert and Rose-Marie Chagoury School of Medicine, Lebanese American University, Beirut P.O. Box 36, Lebanon; yasir_waheed_199@hotmail.com; 13 Department of Virology, Postgraduate Institute of Medical Education and Research, Chandigarh 160012, India; prakasini.satapathy@gmail.com; 14 Escuela de Medicina, Universidad César Vallejo, Trujillo 13007, Peru; 15 Clinical Epidemiology and Biostatistics Program, Faculty of Health Sciences, Universidad Científica del Sur, Lima 4861, Peru; arodriguezmo@cientifica.edu.pe; 16 Grupo de Investigación Biomedicina, Faculty of Medicine, Fundación Universitaria Autónoma de las Américas-Institución Universitaria Visión de las Américas, Pereira 660003, Risaralda, Colombia; 17 Department of Microbiology, Institute of Medicine, Tribhuvan University Teaching Hospital, Kathmandu 44600, Nepal; ranjitsahdoc@gmail.com; 18 Department of Microbiology, Dr. D.Y. Patil Medical College, Hospital and Research Centre, Dr. D.Y. Patil Vidyapeeth, Pune 411018, India; 19 Department of Public Health Dentistry, Dr. D.Y. Patil Dental College and Hospital, Dr. D.Y. Patil Vidyapeeth, Pune 411018, Maharashtra, India

**Keywords:** avian influenza, gene ontology, H5N1 virus, hub genes, KEGG pathways

## Abstract

Introduction: H5N1 is a highly pathogenic avian influenza virus that can infect humans and has an estimated fatality rate of 53%. As shown by the current situation of the COVID-19 pandemic, emerging and re-emerging viruses such as H5N1 have the potential to cause another pandemic. Thus, this study outlined the hub genes and pathways associated with H5N1 infection in humans. Methods: The genes associated with H5N1 infection in humans were retrieved from the NCBI Gene database using “H5N1 virus infection” as the keyword. The genes obtained were investigated for protein–protein interaction (PPI) using STRING version 11.5 and studied for functional enrichment analysis using DAVID 2021. Further, the PPI network was visualised and analysed using Cytoscape 3.7.2, and the hub genes were obtained using the local topological analysis method of the cytoHubba plugin. Results: A total of 39 genes associated with H5N1 infection in humans significantly interacted with each other, forming a PPI network with 38 nodes and 149 edges modulating 74 KEGG pathways, 76 biological processes, 13 cellular components, and 22 molecular functions. Further, the PPI network analysis revealed that 33 nodes interacted, forming 1056 shortest paths at 0.282 network density, along with a 1.947 characteristic path length. The local topological analysis predicted IFNA1, IRF3, CXCL8, CXCL10, IFNB1, and CHUK as the critical hub genes in human H5N1 infection. Conclusion: The hub genes associated with the H5N1 infection and their pathways could serve as diagnostic, prognostic, and therapeutic targets for H5N1 infection among humans.

## 1. Introduction

The highly pathogenic avian influenza (HPAI) H5N1 virus, belonging to the Orthomyxoviridae family, has significant potential to cause a devastating pandemic [1]. The H5N1 infection was initially confined to poultry but gradually became a severe threat among humans over time [2]. In 1997, the H5N1 virus was first reported to breach the human–avian barrier, causing an outbreak among 18 patients, 6 of whom died, in Hong Kong, China [3]. Additionally, it re-emerged in 2003 in China, and the virus has become entrenched in poultry and continues to evolve in parts of Asia, Africa, and the Middle East [4]. Globally, 868 cases of human infection with the H5N1 virus have been documented, with a fatality rate of 53% [5]. The mortality rate was predominantly among patients 10–19 years of age, and the median age of patients with H5N1 infection was approximately 18 years [6]. Transmission of H5N1 occurs predominantly via exposure to infected poultry, but there have been a few instances of suspected human–human transmission. Limited data on the incubation period reveals that illness occurs within seven days of exposure to infected poultry. In human–human transmission, it appears to take 3–5 days [7]. In addition, it has been revealed that the combination of two surface proteins, haemagglutinin (HA) and Neuraminidase (NA), plays a crucial role in the pathogenicity of the H5N1 virus [8]. HA is a surface glycoprotein that virus particles utilise to bind to the cell surface receptors. The posttranslational cleavage of polyprotein percussor (HA0) plays a crucial role in viral infectivity [9]. The NA protein facilitates the mobility of virions by removing the viral HA during the entry and release from the cell. In addition, the balance between the HA and NA is of great importance, as it is found that sufficient HA activity is necessary for the binding of the virus and NA activity to allow the release of viral progeny [10]. H5N1 infection symptoms may range from moderate (fever, malaise, cough, sore throat) to severe illness (shortness of breath, pneumonia, acute respiratory distress syndrome) [11,12]. However, there is an evolving concern that the H5N1 virus may undergo evolutionary changes via mutation (antigenic drift), re-assortment (antigenic shift), and, in rare instances, recombination, which allows efficient human–human transmission, potentially leading to a new influenza pandemic [13,14]. According to phylogenetic analyses of H5 haemagglutinin (HA), HPAI H5N1 viruses have evolved into ten antigenically different clades, which have resulted in antigenic mismatches when choosing the vaccination strain(s). Further, there is no evidence that any vaccination strains can produce cross-protective immunity against a variety of H5N1 clades and subclades. Thus, for the development of an H5N1 vaccine, the World Health Organisation (WHO) has recommended 32 candidate vaccine viruses from different clades and subclades of the virus that might be effective in controlling infection by various H5N1 clades/subclades [15]. The ideal H5N1 vaccine should provide cross-protection against viruses from many clades and must be able to trigger a robust immune response with minimal antigen. To show this ideal characteristic, the vaccine must activate mechanisms that successfully prime and induce adaptive immunity in the naive host. However, due to the fact that vaccine effectiveness varies with age, race, and health state, immunological priming is challenging [16].

Designing a vaccine is a critical and complex task. The traditional vaccine development process is complicated, time-consuming, and associated with a high failure rate at an advanced stage. The scientific community’s attention has recently been directed towards the unusual need for quick vaccine design because of previous epidemic outbreaks, including the COVID-19 pandemic, the influenza virus, SARS, and, more recently, the virus that causes the Zika disease. This has encouraged the application of computational tools to investigate suitable vaccine candidates [17]. Currently, the vaccines against H5N1virus available on the market primarily target the haemagglutinin surface antigens. However, by the time a vaccine is released on the market, a new strain of haemagglutinin develops resistance [18]. Nevertheless, the systems biology approach can provide a powerful platform for identifying potential hub genes that can target surface antigens and adequately understand relative signalling pathways to develop effective vaccines [18,19]. The integration of computational methods has drastically reduced the vaccine development process from 15–20 years to 2–3 years and further allows the screening of multiple novel candidates [17]. The use of computational methods in immunology has led to the development of a new vaccine design paradigm. Immunoinformatics can be used in various vaccine development approaches, such as personalized vaccination, pathogens with antigenic variability, and ERID [20].

Currently, the healthcare system is still recuperating from the havoc created by the COVID-19 pandemic. Furthermore, there have been reports of the appearance of novel variants across the globe, including the resurgence of the Marburg virus in Ghana [21], the Visceral leishmaniasis outbreak in Kenya [22], the camel virus in Qatar [23], and the advent of rabies in Africa [24]. In a similar vein, Spanish public health officials reported the discovery of (H5N1) in two poultry workers on a single farm following a confirmed epidemic in poultry on 20 September 2022 [25]. Henceforth, the H5N1 virus poses a significant threat to public health due to its capacity to infect a wide range of hosts and the lack of information on human transmission.

With regard to this, we carried out a systems biology-based analysis of the genes associated with H5N1 infection in humans from the NCBI Gene database to outline the hub genes and regulated pathways that drive H5N1 infection in humans, which could serve as diagnostic or therapeutic targets for the disease control.

## 2. Material and Methods

### 2.1. Data Acquisition

The data for the genes involved in H5N1 virus infection in humans were retrieved from the NCBI Gene database (https://www.ncbi.nlm.nih.gov/gene/ (accessed on 1 December 2022) using the keyword “H5N1 virus infection” [26]. The search details for the keyword used were: ((“H5N1 subtype” [Organism] OR H5N1 [All Fields]) AND virus [All Fields] AND infection [All Fields]) AND alive [prop].

### 2.2. Construction of Protein–Protein Network and Cluster Analysis

The retrieved genes from NCBI Gene associated with human H5N1 infection were used to construct a protein–protein interaction (PPI) network. The PPI network was constructed using the STRING 11.5 database (https://string-db.org/ accessed on 1 December 2022) [27]. The PPI network was constructed using the full STRING network as the network type (i.e., the edges indicate both functional and physical protein associations), the evidence as network edges (i.e., the colour of the edges indicates the type of interaction) at a minimum interaction score (i.e., medium confidence of 0.400). The nodes in the PPI network represent the protein encoded by the particular gene. Further, node colour (red or white) and node content (empty or filled) were used to represent different nodes.

The PPI network was further analysed for the cluster of the genes via the k-means clustering method of STRING 11.5 for three distinct clusters. The edges between the three clusters were represented by a dotted line, and the proteins in all the clusters have a unique colour. 

### 2.3. Functional Enrichment Analysis of Genes

The genes associated with the human H5N1 infection were further enriched to identify the modulated Kyoto Encyclopedia of Genes and Genomes (KEGG) pathways and Gene Ontology (GO) terms (biological process, cellular components, and molecular function) using the Database for Annotation, Visualization, and Integrated Discovery 2021 (DAVID 2021) (https://david.ncifcrf.gov/ accessed on 1 December 2022) [28,29]. Fisher’s exact test was used to analyse the significance of the enrichment. The enrichment was considered significant if the *p*-value < 0.05.

### 2.4. Identification of Hub Genes for H5N1 Infection

The PPI network for the H5N1 genes was visualised and analysed in Cytoscape 3.7.2. The PPI network was analysed by treating it as undirected and visualised, setting the node size “low values to small size” and “low values to bright colours” based on the average shortest path length for both settings. Further, the network was investigated for the top ten hub genes involved in H5N1 infection in humans using the cytoHubba plugin for Cytoscape 3.7.2 via local topological analysis methods, such as Degree, MCC, DNMC, and MNC. The common genes between these four topological analyses were considered the key regulators of H5N1 infection among humans.

## 3. Results

### 3.1. Data Acquisition

The search resulted in a total of 72 genes, out of which only 39 genes were relevant to Homo sapiens (Supplementary Table S1). In contrast, the other genes were for the Mus musculus (23 genes), Influenza A virus (8 genes), and Gallus gallus (2 genes).

### 3.2. Construction of Protein-Protein Network and Cluster Analysis

**Figure 1:**
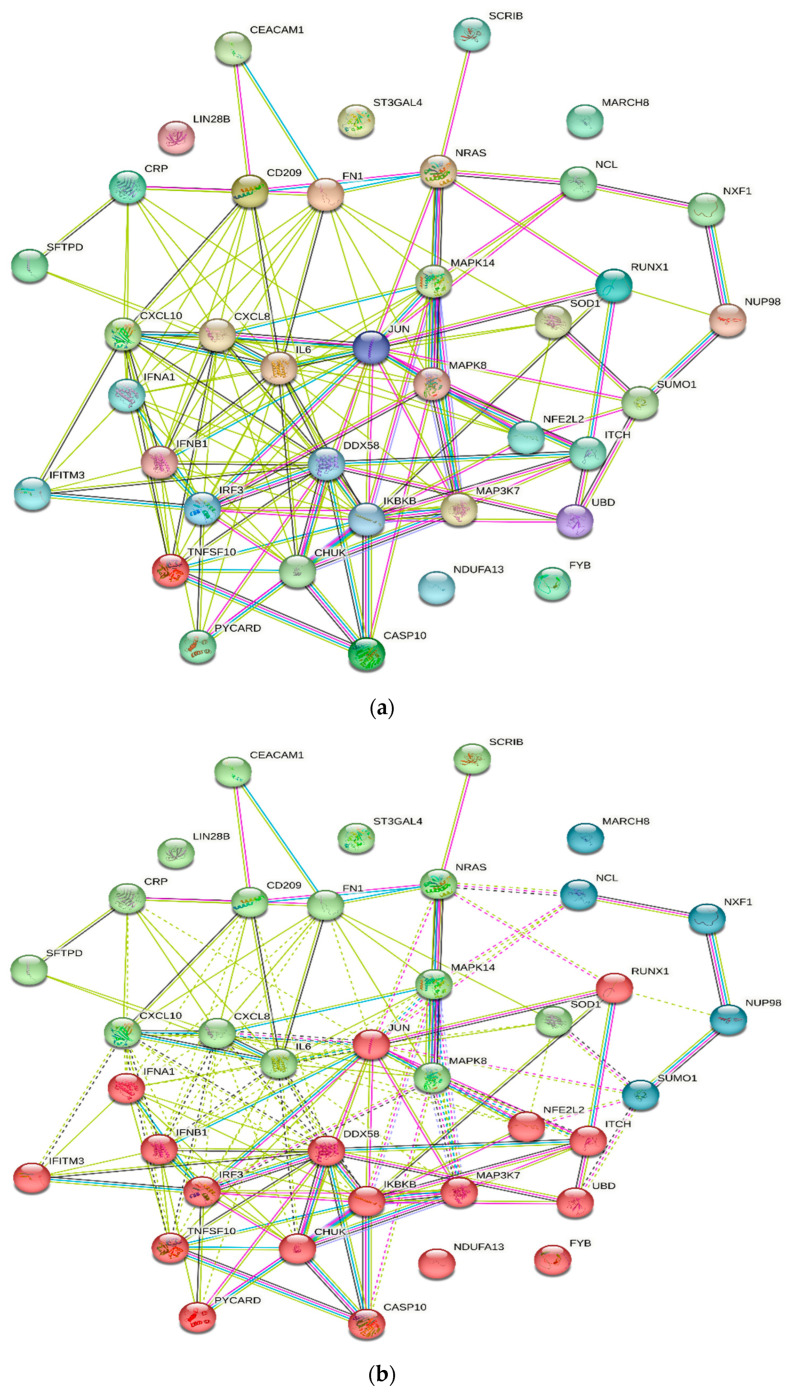
(**a**) Protein–protein interaction of genes associated with H5N1 infection in humans. Nodes represent proteins and edges represent protein–protein associations. (**b**) Cluster analysis of protein–protein interaction. First cluster (red colour) consists of 18 nodes; second cluster (green colour) consists of 15 nodes; and third cluster (blue colour) consists of 5 nodes.

The PPI network of human genes associated with H5N1 infection obtained from the STRING database consists of a total of 38 nodes and 149 edges with a 7.84 average node degree and 0.483 average local clustering coefficient. The PPI was found to be significant, with the *p*-value < 1.0 × 10^−16^. The protein–protein interactions of the H5N1 infection-associated genes in humans consists of nodes and edges in the network which represent proteins and protein–protein association, respectively. These interactions were based on node colour, i.e., coloured nodes (query proteins and first shell of interactions), white nodes (second shell of interactions), and node content, i.e., empty node protein of unknown 3D structure), and filled nodes (some 3D structure is known). Further, it was also based on the known interactions (curated databases and experimentally determined), predicted interactions (gene neighbourhood, gene fusions, and gene co-occurrence), and others (text mining, co-expression, and protein homology) (Figure 1a, Supplementary Table S2).

Further, the cluster analysis of the PPI network via k-means clustering methods resulted in three distinct clusters, interconnected by dotted lines (Figure 1b). The first cluster (red colour cluster) of genes was formed by the interactions of 18 different genes: CASP10, CHUK, DDX58, FYB, IFITM3, IFNA1, IFNB1, IKBKB, IRF3, ITCH, JUN, MAP3K7, NDUFA13, NFE2L2, PYCARD, RUNX1, TNFSF10, and UBD. Similarly, the second cluster (green colour) consists of the interactions of 15 different genes: CD209, CEACAM1, CRP, CXCL10, CXCL8, FN1, IL6, LIN28B, MAPK14, MAPK8, NRAS, SCRIB, SFTPD, SOD1, and ST3GAL4, followed by the third cluster (blue colour) consisting of the interactions of 5 different genes: Mar-08, NCL, NUP98, NXF1, and SUMO1.

### 3.3. Functional Enrichment Analysis of Genes

#### 3.3.1. KEGG Pathways

**Figure 2:**
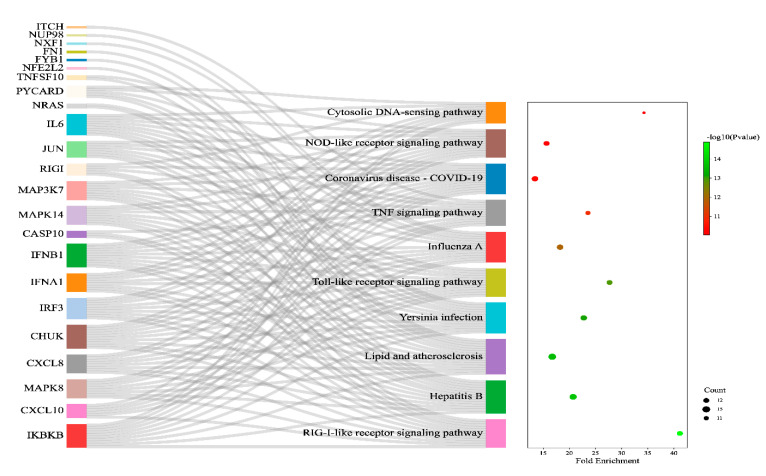
Sankey and dot plot for top ten KEGG pathways involved in H5N1 infection. The associated protein–pathway interactions with respective fold enrichment, gene count, and *p*–value.

The enriched genes associated with H5N1 infection in humans were found to modulate a total of 86 KEGG pathways, of which 74 pathways were significantly (*p*-value < 0.05) modulated by the enrichment of the genes (Supplementary Table S3). Out of the 74 significantly modulated KEGG pathways, the top ten pathways were the RIG-I-like receptor signalling pathway, Hepatitis B, lipid and atherosclerosis, Yersinia infection, Toll-like receptor signalling pathway, Influenza A, TNF signalling pathway, Coronavirus disease—COVID-19, NOD-like receptor signalling pathway, and cytosolic DNA-sensing pathway (Figure 2).

#### 3.3.2. Gene Ontology

Enriching genes associated with H5N1 infection in humans significantly modulated 76 biological processes, 13 cellular components, and 22 molecular functions (Supplementary Table S4).

The top ten modulated biological processes were a cellular response to the virus, positive regulation of the apoptotic process, defence response to the virus, inflammatory response, cellular response to cadmium ion, positive regulation of I-kappaB kinase/NF-kappaB signalling, cellular response to lipopolysaccharide, positive regulation of gene expression, cellular response to tumour necrosis factor, and positive regulation of transcription from the RNA polymerase II promoter, of which the cellular response to the virus was a highly significant (3.06018540743041E-10) biological process modulated via the regulation of eight different genes: CXCL10, IL6, CHUK, IRF3, IFNA1, IFNB1, MIR21, and MAPK14 (Table 1).

Further, the top ten modulated cellular components were the extracellular region, IkappaB kinase complex, extracellular space, nucleoplasm, cytosol, nuclear pore, cytoplasm, CD40 receptor complex, nuclear inclusion body, and macromolecular complex, of which the extracellular region was the highly significant (6.79 × 10^−5^) modulated cellular component via the regulation of 14 different genes: CRP, CXCL8, IFNA1, IFNB1, SFTPD, FN1, MAPK14, SOD1, PYCARD, CXCL10, IL6, CD209, TNFSF10, and ST3GAL4 (Table 2).

Additionally, the top ten modulated molecular functions were identical protein binding, protein binding, CXCR chemokine receptor binding, MAP kinase activity, enzyme binding, ubiquitin-like protein ligase binding, IkappaB kinase activity, cytokine activity, protein serine/threonine kinase activity, and scaffold protein binding, of which identical protein binding was the highly significant (8.73 × 10^−5^) modulated molecular function via the regulation of 13 different genes: CRP, JUN, SFTPD, FN1, SOD1, PYCARD, IKBKB, CEACAM1, IRF3, NCL, TNFSF10, MAP3K7, and RIGI (Table 3). 

### 3.4. Identification of Hub Genes for H5N1 Infection

**Figure 3:**
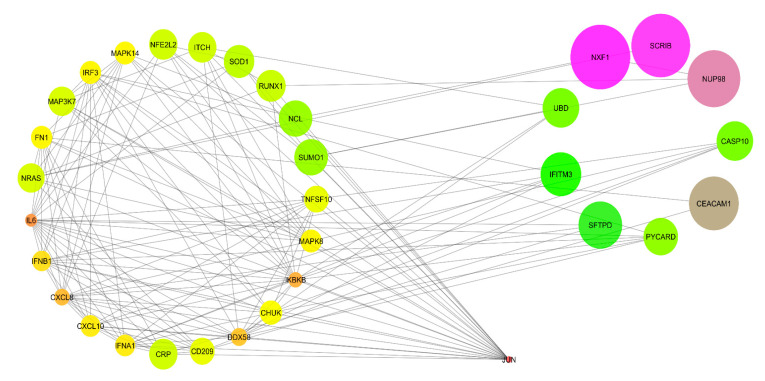
Visualisation and analysis of protein–protein interactions. Undirected analysis and visualised node size “*low values to small size*” and “*low values to bright colours*” based on average shortest path length.

**Figure 4:**
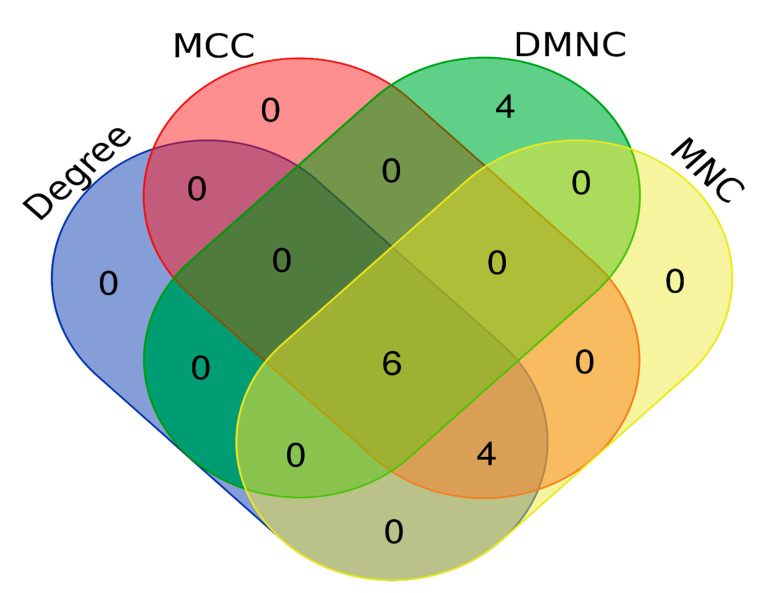
Common hub genes among local topological methods such as MCC, MNC, DMNC, and Degree.

The PPI network from STRING was imported into Cytoscape 3.7.2 for visualisation and analysis. The visualisation was made based on the average shortest path length for the mode size and node colour. The PPI network analysis revealed that a total of 33 nodes interacted with each other, forming 1056 shortest paths at 0.282 network density along with a 1.947 characteristic path length (Figure 3). Further, the PPI network was analysed using local topological analysis methods, such as MCC, MNC, DMNC, and Degree, to identify the top ten hub genes involved in H5N1 infection in humans (Supplementary Table S5 & Figure S1). Out of the total of 14 different hub genes identified via the local topological analysis; we considered the intersection of the hub genes among all the local topological methods as the key regulator of H5N1 infection in humans. The common hub genes among all the local topological methods were IFNA1, IRF3, CXCL8, CXCL10, IFNB1, and CHUK (Table 4, Figure 4). Out of the six hub genes, IFNA1, IFNB1, IRF3, and CHUK fall in the category of the first cluster (red colour cluster), whereas CXCL8 and CXCL10 belong to the second cluster (green colour cluster). Thus, targeting cluster 1 and cluster 2 is enough to cover the hub proteins involved in human H5N1 infection.

## 4. Discussion

H5N1 virus infection is commonly observed in birds/poultry but has gradually become deadly among Homo sapiens. The H5N1 virus can infect the pulmonary (respiratory tract and lungs) and extrapulmonary sites (brain, lymph node, spleen, liver, and intestine, among others). H5N1 infection in the host cell begins with the entry of the H5N1 virus. The viral entry to the host cell is accompanied by the binding of the haemagglutinin (HA) surface protein to the sialic acid receptor (α2–3 sialic acid), which undergoes the endocytosis process. Later, the endosome membrane fuses with the viral envelope, with the help of M1 and M2 proteins promoting the release of its genetic content into the cytoplasm of the host cell, which moves to the nucleus of the host cell, where it starts the RNA replication, transcription, and translation in the presence of the ribonucleoprotein complex (PB1, PB2, PA, PB1-F2, and NP). Finally, the new virions formed bud off from the host cell and are detached with the help of neuraminidase (NA) [2]. The H5N1 infection is still novel and poorly understood regarding its pathogenesis and treatment options, primarily concerning its genetic aspects. We found that crucial hub genes, such as IFNA1, IRF3, CXCL8, CXCL10, IFNB1, and CHUK, were associated with the human H5N1 infection, which might serve as a diagnostic, prognostic, or therapeutic target. 

Immediately after an H5N1 virus infection, our body responds to the infection via the activation of type I interferons (INF), such as INFA and INFB. The transcription of these INF occurs via the activation of the RIG-I signalling pathway. The activation of the RIG-I signalling pathway drives the activation of INF through a series of intermediate signalling molecules, such as IPS1, IRF3, and ISGs. Although the major reason behind the production of these INF (INFA and INFB) is to mediate the antiviral action via the JAK-STAT signalling pathway, influenza viruses often manage to escape it with the help of non-structural protein (NS1) [30]. This supports the finding of our study; we report that interferons such as INFA1 and INFB1 are the hub genes associated with human H5N1 infections. Further, we observed regulation of both IFNA1 and IFNB1 modulated several pathways, such as the RIG-I-like receptor signalling pathway, Hepatitis B, lipid and atherosclerosis, Toll-like receptor signalling pathway, Influenza A, Coronavirus disease—COVID-19, NOD-like receptor signalling pathway, and cytosolic DNA-sensing pathway, whereas regulation of IFNB1 modulated Yersinia infection and the TNF signalling pathway.

Interleukin-6 (IL6) is a cytokine which plays a prominent role in immune and inflammatory responses involved in the activation of T-cells [31]. The H5N1 infection was previously reported to induce IL6 from the bronchial epithelial cell line [32]. A study by Chan et al. observed an upregulation of IL6 expression in bronchial cells, contributing synergistically to the pathogenesis of H5N1 infection [33]. IL6 was found to modulate several pathways related to H5N1 infection, such as the Toll-like receptor signalling pathway, Influenza A, TNF signalling pathway, Coronavirus disease—COVID-19, NOD-like receptor signalling pathway, and cytosolic DNA-sensing pathway, in our study. CXCL10/IP-10 is a cytokine belonging to the CXC chemokine family. It can exert various biological functions such as the promotion of cell growth, induction of apoptosis, proliferation, and angiogenesis in infectious diseases. IP-10 is a macrophagic chemoattractant and exacerbates the inflammatory response by further recruitment of circulating leukocytes [34]. The strong induction of IP-10 could explain the prominent macrophagic infiltrates in the lungs of patients with H5N1 infection [35]. We report that both CXCL8 and CXCL10 modulate the pathways associated with influenza infection, such as the Toll-like receptor signalling pathway, Influenza A, and the RIG-I-like receptor signalling pathway. Further, CXCL10 also modulates the TNF signalling pathway and cytosolic DNA-sensing pathway in our study. Similarly, we also observed that CXCL8 modulated the NOD-like receptor signalling pathway. DDX58, also known as RIG1, belongs to the RIG-1-like receptor family, which is a cytoplasmic viral RNA receptor with 925 residues [36]. However, the role of the RIG1 receptor signalling pathway in the progress of Influenza A virus infection has been widely discussed. C-Jun is a downstream molecule of the JNK signalling pathway, which acts as a critical factor of activator protein AP-1 and may participate in the viral infection establishment process. A study showed that the downregulation of c-Jun significantly suppressed viral replication but also mitigated the expression of proinflammatory cytokines [37]. In our study, we found *JUN* modulates several pathways, such as the Toll-like receptor signalling pathway, TNF signalling pathway, Coronavirus disease—COVID-19, and NOD-like receptor signalling pathway.

The functional enrichment analysis was shown to modulate pathways such as the RIG-I-like receptor signalling pathway, Hepatitis B, lipid and atherosclerosis, Yersinia infection, Toll-like receptor signalling pathway, Influenza A, TNF signalling pathway, Coronavirus disease—COVID-19, NOD-like receptor signalling pathway, and cytosolic DNA-sensing pathway in our study. The initiation of the innate immune response to infection is mediated by pattern recognition receptors, such as Toll-like receptors, NOD-like receptors, and RIG-1-like receptors, and initiates antiviral signalling cascades [38]. TLRs are the first receptors to recognise influenza virus A infection. Predominantly, TLR7, TLR3, and TLR4 are involved in sensing viral infections. TLR7 recognises the sRNA; TLR3 identifies the dsRNA; whereas TLR4 recognises the viral glycoproteins [39]. The Toll-like receptor signalling pathway is activated via MyD88, which subsequently can activate tumour necrosis factor (TNF) receptor-associated factor 6 (TRAF6) and activates nuclear factor kappa-light-chain-enhancer of activated B cells (NF-κB) through IRAKs, TRAF6, TAK1, and IKK complex, resulting in the induction of inflammatory cytokines [40]. Several studies have reported dysregulation of proinflammatory cytokines and chemokines in Influenza A virus infection. In our study, we found the Toll-like signalling pathway to be modulated via the regulation of 12 different genes: IKBKB, CXCL10, IL6, JUN, MAPK8, CXCL8, CHUK, IRF3, IFNA1, IFNB1, MAPK14, and MAP3K7, which could ultimately lead to the dysregulation of proinflammatory mediators [2]. Further, the TNF signalling pathway and NF-kappa B signalling pathway were found to be affected by the regulation of 11 different genes: IKBKB, CXCL10, IL6, ITCH, JUN, MAPK8, CHUK, IFNB1, CASP10, MAPK14, and MAP3K7, and 5 different genes: IKBKB, CXCL8, CHUK, MAP3K7, and RIGI, respectively. The activation of RIG-1 receptors by the dsRNA of a virus leads to confirmational changes and caspase activation and recruitment domain multimerization. It then becomes associated with the mitochondrial antiviral signalling adaptor, then transduces signals to TRAF3 and TBK1, leading to the translocation of IRF-3, IRF-7, and the activation of NF-κB. This process leads to increased activity of type 1 interferon proinflammatory factors, ultimately delaying viral replication [41]. However, Influenza A virus is known to interfere with the RIG-1 signalling pathway majorly via its multifunctional virulence factor, i.e., nonstructural protein 1(NS1). NS1 protein blocks the recognition of viral ssRNA by the RIG-1 receptor and ultimately inhibits the activation of proinflammatory cytokines [39]. Among the NOD-like receptors, the most-concerned receptor is NLRP3, which is simulated by the virus, and the protein starts oligomerization, subsequently binding to ASC and Caspase-1, and facilitating the maturation of pro-IL-1β and cell death [42]. A study by Teijaro et al. reported that NLRP3 inflammasome is associated with a cytokine storm and results in high mortality in IAV infections [43]. We observed that RIG-I-like signalling pathways and NOD-like receptor signalling pathways were modulated via the regulation of IKBKB, CXCL10, MAPK8, CXCL8, CHUK, IRF3, IFNA1, IFNB1, CASP10, MAPK14, MAP3K7, RIGI, and PYCARD, IKBKB, IL6, JUN, MAPK8, CXCL8, CHUK, IRF3, IFNA1, IFNB1, MAPK14, and MAP3K7, respectively.

The MAPK signalling pathway is known to play a crucial role in cell proliferation, differentiation, migration, and immune response [44]. A study by Hui et al. showed that the H5N1 virus activated the MAPK signalling pathway [45]. An accumulation of haemagglutinin (HA) upregulated the Raf/MEK/ERK cascade within the infected cells for efficient export of viral RNP and the viral protein expression [46]. We found that the MAPK signalling pathway was modulated in H5N1 infections via the regulation of seven different genes: IKBKB, JUN, NRAS, MAPK8, CHUK, MAPK14, and MAP3K7. The aberrant presence of DNA in the cytoplasm is usually a result of microbial infection and elicits a robust immune response via the DNA-sensing signalling pathway [47]. Similarly, our study proved the DNA-sensing signalling pathway to be modulated during H5N1 infection via the regulation of nine different genes: PYCARD, IKBKB, CXCL10, IL6, CHUK, IRF3, IFNA1, IFNB1, and RIGI. In the DNA-sensing signalling pathway, cyclic GMP–AMP synthase(cGAS) is activated during the binding of dsDNA and is converted to cGAMP, which in turn activates stimulators of interferon genes (STING) to activate proinflammatory cytokines via IRF3 and NF-kB [48]. Infection with IAV causes significant upper and lower lung parenchymal cell death through the apoptosis process [49], which corroborates the results of a study by Daidoji et al. [50]. Apoptosis occurs mainly by the interaction of IAV protein PB1-F2 with VDAC1 and ANT3 to promote the activation of CASP9 and CASP3, and the release of pro-apoptotic molecules [51].

Although the vaccines for H5N1 are designed to counteract the antigen, such as neuraminidase and the surface glycoprotein haemagglutinin, the effectiveness of vaccines to control emerging new strains is still a major drawback, which occurs because of the antigenic shift and drift. As the surface glycoprotein stimulates the immune system, the protein involved can be sequenced using a bioinformatics approach, which could provide a novel vaccine candidate that can accommodate antigenic shift/drift [20]. It has been found that immunosenescence plays a vital role in the response of the body to influenza vaccination. This necessitates the development of strong quadrivalent seasonal influenza vaccines, which can be achieved via an understanding of the transcriptome mechanism underlying the quadrivalent influenza vaccine’s ability to induce immunity, as well as investigating possible hub genes and signalling pathways. Thus, novel system vaccinology is more focused towards the interaction between the vaccine and host immunity [19]. Hence, it is important to understand the possible hub genes and signalling pathways involved in H5N1 infection in human hosts for the future vaccinology approach. Further, integrating omics data, such as genomics, transcriptomics, proteomics, and immunomics, along with computational modelling approaches will drive vaccine design and development [52]. Hence, the hub genes (IFNA1, IRF3, CXCL8, CXCL10, IFNB1, and CHUK) identified in the present study can serve as the basis for the development of a vaccine or theranostic biomarker for H5N1 infection among human hosts. Although the current study identified the key hub protein associated with the H5N1 infection, our study lacks the validation of the hub proteins via in vitro (ELISA/Western blotting) and in vivo approaches, which provide the scope for further investigation of these hub genes for their implication in vaccine development.

## 5. Conclusions

We conclude that IFNA1, IRF3, CXCL8, CXCL10, IFNB1, and CHUK are the key hub genes involved in the H5N1 human infection, which makes a major contribution towards the inflammation or cytokine storm leading to the prognosis of the disease and critical clinical outcomes. Thus, these hub genes and their pathways could be used as diagnostic, prognostic, or therapeutic targets, or in vaccine development to tackle the H5N1 infection.

## Figures and Tables

**Table 1:** Top Ten Biological Processes in H5N1 Infection. Colour map indicates; Red colour: Low value, Yellow colour: Medium value, Green colour: High value.

Gene Ontology Terms	Genes	Count	*p*-Value	Fold Enrichment	FDR
Cellular response to virus	CXCL10, IL6, CHUK, IRF3, IFNA1, IFNB1, MIR21, MAPK14	8	3.06 × 10^−10^	46.05366726	2.19 × 10^−7^
Positive regulation of the apoptotic process	PYCARD, IL6, JUN, MAPK8, UBD, TNFSF10, MIR21, SCRIB, SOD1	9	2.00 × 10^−7^	13.58442777	7.15 × 10^−5^
Defence response to virus	PYCARD, IFITM3, IL6, ITCH, IRF3, IFNA1, IFNB1, RIGI	8	3.13 × 10^−7^	16.99834929	7.46 × 10^−5^
Inflammatory response	PYCARD, CRP, IKBKB, CXCL10, IL6, ITCH, CXCL8, CHUK, NFE2L2	9	1.12 × 10^−6^	10.81478715	1.76 × 10^−4^
Cellular response to cadmium ion	JUN, MAPK8, CHUK, SUMO1, SOD1	5	1.23 × 10^−6^	60.37523452	1.76 × 10^−4^
Positive regulation of I-kappaB kinase/NF-kappaB signalling	IKBKB, CHUK, IRF3, CASP10, UBD, TNFSF10, MAP3K7	7	1.89 × 10^−6^	18.04967949	2.00 × 10^−4^
Cellular response to lipopolysaccharide	PYCARD, CXCL10, IL6, MAPK8, CXCL8, MIR21, MAPK14	7	1.95 × 10^−6^	17.95615783	2.00 × 10^−4^
Positive regulation of gene expression	CRP, IL6, MAPK8, CXCL8, FN1, MIR21, MAPK14, RIGI, NFE2L2	9	5.95 × 10^−6^	8.635062612	5.32 × 10^−4^
Cellular response to tumour necrosis factor	PYCARD, IKBKB, CXCL8, CHUK, MAPK14, NFE2L2	6	9.48 × 10^−6^	20.34562698	7.54 × 10^−4^
Positive regulation of transcription from RNA polymerase II promoter	IKBKB, CXCL10, IL6, JUN, CHUK, IRF3, IFNB1, NCL, MAPK14, RIGI, NFE2L2, RUNX1	12	1.33 × 10^−5^	4.934321492	9.55 × 10^−4^
**Colour map**			Low	Medium	High

**Table 2:** Top Ten Cellular Components in H5N1 Infection. Colour map indicates; Red colour: Low value, Yellow colour: Medium value, Green colour: High value.

Gene Ontology Terms	Genes	Count	*p*-Value	Fold Enrichment	FDR
Extracellular region	CRP, CXCL8, IFNA1, IFNB1, SFTPD, FN1, MAPK14, SOD1, PYCARD, CXCL10, IL6, CD209, TNFSF10, ST3GAL4	14	6.79 × 10^−5^	3.466994255	0.004652
IkappaB kinase complex	PYCARD, IKBKB, CHUK	3	6.94 × 10^−5^	225.956044	0.004652
Extracellular space	CRP, CXCL10, IL6, CXCL8, IFNA1, IFNB1, MIR141, SFTPD, TNFSF10, FN1, MIR21, SOD1	12	5.53 × 10^−4^	3.264586806	0.02468
Nucleoplasm	NDUFA13, JUN, CHUK, SCRIB, MAPK14, SOD1, RUNX1, PYCARD, NXF1, ITCH, MAPK8, IRF3, SUMO1, UBD, NCL, NUP98, NFE2L2	17	0.001021	2.261651041	0.034211
Cytosol	JUN, FYB1, CHUK, MAPK14, SOD1, PYCARD, IKBKB, NXF1, ITCH, NRAS, MAPK8, IRF3, SUMO1, CASP10, UBD, NUP98, MAP3K7, RIGI, NFE2L2	19	0.00521	1.818037135	0.139638
Nuclear pore	NXF1, SUMO1, NUP98	3	0.012799	17.00744417	0.280061
Cytoplasm	NDUFA13, CHUK, MIR21, MAPK14, SOD1, PYCARD, IKBKB, NXF1, ITCH, MAPK8, IRF3, CASP10, CD209, UBD, NCL, MARCHF8, RIGI, NFE2L2	18	0.01463	1.701964463	0.280061
CD40 receptor complex	IKBKB, CHUK	2	0.020147	95.86013986	0.337459
Nuclear inclusion body	NXF1, NUP98	2	0.023767	81.11242604	0.353867
Macromolecular complex	PYCARD, IFITM3, ITCH, FYB1, SOD1	5	0.039911	3.749863224	0.480924
**Colour map**			Low	Medium	High

**Table 3:** Top Ten Molecular Functions in H5N1 Infection. Colour map indicates; Red colour: Low value, Yellow colour: Medium value, Green colour: High value.

Gene Ontology Terms	Genes	Count	*p*-Value	Fold Enrichment	FDR
Identical protein binding	CRP, JUN, SFTPD, FN1, SOD1, PYCARD, IKBKB, CEACAM1, IRF3, NCL, TNFSF10, MAP3K7, RIGI	13	8.73 × 10^−5^	3.66958382	0.012314
Protein binding	IFITM3, CRP, NDUFA13, CXCL8, IFNA1, IKBKB, PYCARD, NXF1, NRAS, MAPK8, SUMO1, CASP10, UBD, TNFSF10, MARCHF8, MAP3K7, JUN, FYB1, CHUK, IFNB1, SFTPD, FN1, SCRIB, MAPK14, RUNX1, SOD1, CXCL10, ITCH, IL6, CEACAM1, IRF3, CD209, NCL, NUP98, RIGI, NFE2L2	36	2.37 × 10^−4^	1.387409468	0.01641
CXCR chemokine receptor binding	CXCL10, ITCH, CXCL8	3	4.08 × 10^−4^	96.76410256	0.01641
MAP kinase activity	MAPK8, MAPK14, MAP3K7	3	4.66 × 10^−4^	90.71634615	0.01641
Enzyme binding	PYCARD, JUN, MAPK8, SUMO1, FN1, MAPK14	6	0.001053	7.443392505	0.024908
Ubiquitin-like protein ligase binding	ITCH, JUN, SUMO1	3	0.00106	60.4775641	0.024908
IkappaB kinase activity	IKBKB, CHUK	2	0.00603	322.5470085	0.118703
Cytokine activity	IL6, IFNA1, IFNB1, TNFSF10	4	0.006735	10.07959402	0.118703
Protein serine/threonine kinase activity	IKBKB, MAPK8, CHUK, MAPK14, MAP3K7	5	0.007755	6.17118001	0.119996
Scaffold protein binding	IKBKB, CHUK, MAP3K7	3	0.00851	21.03567447	0.119996
**Colour map**			Low	Medium	High

**Table 4:** Hub Genes via the Local Topological Analysis Methods.

Local Topological Analysis Method	Gene Count	Hub Gene
MCC, MNC, DMNC, and Degree	6	IFNA1, IRF3, CHUK, CXCL8, IFNB1, CXCL10
MCC, MNC, and Degree	4	IKBKB, JUN, IL6, DDX58
DMNC	4	CRP, TNFSF10, PYCARD, CD209
**Total hub genes**	**14**	

## Data Availability

Not applicable.

## Supplementary Materials

The following are available online at https://www.mdpi.com/article/10.3390/vaccines11071269/s1, Supplementary Table S1: List of Human H5N1 infection associated genes retrieved from the NCBI-gene database, Supplementary Table S2: Details about nodes and edges in PPI network, Supplementary Table S3: KEGG pathways involved in H5N1 infection, Supplementary Table S4: Gene ontology for Human H5N1 infection associated genes, Supplementary Table S5: Top ten hub genes by their rank based on MCC analysis, Supplementary Figure S1: Protein-protein interactions for Local Topological Analysis Methods.
