# Supplementary material for: System Biology Approach to Identify the Hub Genes and Pathways Associated with Human H5N1 Infection
Source: Vaccines (Basel). 2023 Jul 21;11(7):1269. doi: 10.3390/vaccines11071269 (PMC10385284; doi:10.3390/vaccines11071269)
Supplement: Supplementary file 1 [file vaccines-11-01269-s001.zip › vaccines-2335767-supplementary.pdf]

Supplementary Table S1: List of Human H5N1 infection associated genes retrieved from the NCBI-gene database

S. No.	Gene ID	Symbol	Aliases	Description
1.	3569	IL6	BSF-2, BSF2, CDF, HGF, HSF, IFN-beta-2, IFNB2, IL-6	interleukin 6
2.	1401	CRP	PTX1	C-reactive protein
3.	3576	CXCL8	GCP-1, GCP1, IL8, LECT, LUCT, LYNAP, MDNCF, MONAP, NAF, NAP-1, NAP1, SCYB8	C-X-C motif chemokine ligand 8
4.	4780	NFE2L2	HEBP1, IMDDHH, NRF2, Nrf-2	NFE2 like bZIP transcription factor 2
5.	406991	MIR21	MIRN21, hsa-mir-21, miR-21, miRNA21	microRNA 21
6.	6647	SOD1	ALS, ALS1, HEL-S-44, IPOA, SOD, STAHP, hSod1, homodimer	superoxide dismutase 1
7.	1432	MAPK14	CSBP, CSBP1, CSBP2, CSPB1, EXIP, Mxi2, PRKM14, PRKM15, RK, SAPK2A, p38, p38ALPHA	mitogen-activated protein kinase 14
8.	8743	TNFSF10	APO2L, Apo-2L, CD253, TL2, TNLG6A, TRAIL	TNF superfamily member 10
9.	3725	JUN	AP-1, AP1, c-Jun, cJUN, p39	Jun proto-oncogene, AP-1 transcription factor subunit
10.	861	RUNX1	AML1, AML1-EVI-1, AMLCR1, CBF2alpha, CBFA2, EVI-1, PEBP2aB, PEBP2alpha	RUNX family transcription factor 1
11.	2335	FN1	CIG, ED-B, FINC, FN, FNZ, GFND, GFND2, LETS, MSF, SMDCF	fibronectin 1
12.	3627	CXCL10	C7, IFI10, INP10, IP-10, SCYB10, crg-2, gIP-10, mob-1	C-X-C motif chemokine ligand 10
13.	5599	MAPK8	JNK, JNK-46, JNK1, JNK1A2, JNK21B1/2, PRKM8, SAPK1, SAPK1c	mitogen-activated protein kinase 8
14.	4893	NRAS	ALPS4, CMNS, KRAS, N-ras, NCMS1, NS6, NRAS	NRAS proto-oncogene, GTPase
15.	23586	RIGI	DDX58, RIG-I, RIG1, RLR-1, SGMRT2	RNA sensor RIG-I
16.	30835	CD209	CDSIGN, CLEC4L, DC-SIGN, DC-SIGN1, hDC-SIGN	CD209 molecule
17.	3456	IFNB1	IFB, IFF, IFN-beta, IFNB	interferon beta 1
18.	7341	SUMO1	DAP1, GMP1, OFC10, PIC1, SENP2, SMT3, SMT3C, SMT3H3, UBL1	small ubiquitin like modifier 1
19.	3551	IKBKB	IKK-beta, IKK2, IKKB, IMD15, IMD15A, IMD15B, NFKBIKB	inhibitor of nuclear factor kappa B kinase subunit beta
20.	3661	IRF3	IIAE7	interferon regulatory factor 3
21.	4691	NCL	C23, Nsr1	nucleolin
22.	1147	CHUK	BPS2, IKBKA, IKK-alpha, IKK1, IKKA, NFKBIKA, TCF16	component of inhibitor of nuclear factor kappa B kinase complex
23.	3439	IFNA1	IFL, IFN, IFN-ALPHA, IFN-alphaD3, IFNA@, leIF D, IFNA1	interferon alpha 1
24.	634	CEACAM1	BGP, BGP1, BGPI	CEA cell adhesion molecule 1
25.	6441	SFTPD	COLEC7, PSP-D, SFTP4, SP-D	surfactant protein D
26.	6885	MAP3K7	CSCF, FMD2, MEKK7, TAK1, TGF1a	mitogen-activated protein kinase kinase kinase 7
27.	406933	MIR141	MIRN141, mir-141	microRNA 141
28.	83737	ITCH	ADMFD, AIF4, AIP4, NAPP1	itchy E3 ubiquitin protein ligase
29.	29108	PYCARD	ASC, CARD5, TMS, TMS-1, TMS1	PYD and CARD domain containing
30.	4928	NUP98	ADIR2, NUP196, NUP96, Nup98-96	nucleoporin 98 and 96 precursor
31.	10410	IFITM3	1-8U, DSPA2b, IP15	interferon induced transmembrane protein 3

32.	23513	SCRIB	CRIB1, SCRB11, Vartul, oSCRIB, SCRIB	scribble planar cell polarity protein
33.	843	CASP10	ALPS2, FLICE-2, FLICE2, MCH4	caspase 10
34.	10537	UBD	FAT10, GABBR1-3, UBD	ubiquitin D
35.	51079	NDUFA13	B16.6, CDA016, CGI-39, GRIM-19, GRIM19, MC1DN28	NADH:ubiquinone oxidoreductase subunit A13
36.	10482	NXF1	MEX67, TAP	nuclear RNA export factor 1
37.	2533	FYB1	ADAP, FYB, PRO0823, SLAP-130, SLAP130, THC3	FYN binding protein 1
38.	6484	ST3GAL4	CGS23, NANTA3, SAT3, SIAT4, SIAT4C, ST-4, ST3GalA.2, ST3GalIV, STZ, gal-NAc6S	ST3 beta-galactoside alpha-2,3-sialyltransferase 4
39.	220972	MARCHF8	CMIR, MARCH-VIII, MARCH8, MIR, RNF178, c-MIR	membrane associated ring-CH-type finger 8

Supplementary Table S2: Details about nodes and edges in PPI network

**Nodes**	
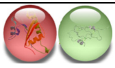 Node color	Colored nodes represent query proteins and first shell of interactions whereas white color nodes represent second shell of interactions.
Node Content 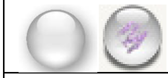	Empty node represents protein of unknown 3D structure whereas filled node represents some 3D structure is known/predicted
**Edges**	
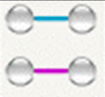 Known Interactions	Azure-blue color between nodes represents curated databases whereas purple color edges represent experimentally determined
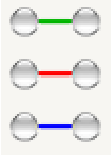 Predicted interactions	Green color edges represent gene neighbourhood, red color edges represent gene fusions and blue color edges represents gene co-occurrence
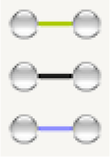 Others	Olive green color edges represent text mining, black color edges represent co-expression, and light blue color edges represent protein homology

Supplementary Table S3: KEGG pathways involved in H5N1 infection

Term	Count	%	P-value	Genes
hsa04622:RIG-I-like receptor signaling pathway	12	30.76923077	1.34E-15	IKBKB, CXCL10, MAPK8, CXCL8, CHUK, IRF3, IFNA1, IFNB1, CASP10, MAPK14, MAP3K7, RIGI
hsa05161:Hepatitis B	14	35.8974359	1.85E-14	JUN, CXCL8, IFNA1, CHUK, IFNB1, MAPK14, IKBKB, IL6, NRAS, MAPK8, IRF3, CASP10, MAP3K7, RIGI
hsa05417:Lipid and atherosclerosis	15	38.46153846	2.67E-14	JUN, CXCL8, IFNA1, CHUK, IFNB1, MAPK14, PYCARD, IKBKB, IL6, NRAS, MAPK8, IRF3, TNFSF10, MAP3K7, NFE2L2
hsa05135:Yersinia infection	13	33.33333333	8.05E-14	JUN, FYB1, CXCL8, CHUK, IFNB1, FN1, MAPK14, PYCARD, IKBKB, IL6, MAPK8, IRF3, MAP3K7
hsa04620:Toll-like receptor signaling pathway	12	30.76923077	1.28E-13	IKBKB, CXCL10, IL6, JUN, MAPK8, CXCL8, CHUK, IRF3, IFNA1, IFNB1, MAPK14, MAP3K7
hsa05164:Influenza A	13	33.33333333	1.17E-12	CXCL8, IFNA1, CHUK, IFNB1, PYCARD, IKBKB, CXCL10, NXF1, IL6, IRF3, TNFSF10, NUP98, RIGI
hsa04668:TNF signaling pathway	11	28.20512821	1.12E-11	IKBKB, CXCL10, IL6, ITCH, JUN, MAPK8, CHUK, IFNB1, CASP10, MAPK14, MAP3K7
hsa05171:Coronavirus disease - COVID-19	13	33.33333333	4.37E-11	JUN, CXCL8, IFNA1, CHUK, IFNB1, MAPK14, IKBKB, CXCL10, IL6, MAPK8, IRF3, MAP3K7, RIGI
hsa04621:NOD-like receptor signaling pathway	12	30.76923077	7.11E-11	PYCARD, IKBKB, IL6, JUN, MAPK8, CXCL8, CHUK, IRF3, IFNA1, IFNB1, MAPK14, MAP3K7
hsa04623:Cytosolic DNA-sensing pathway	9	23.07692308	9.49E-11	PYCARD, IKBKB, CXCL10, IL6, CHUK, IRF3, IFNA1, IFNB1, RIGI
hsa05162:Measles	11	28.20512821	9.80E-11	IKBKB, IL6, JUN, MAPK8, CHUK, IRF3, IFNA1, IFNB1, CD209, MAP3K7, RIGI
hsa05169:Epstein-Barr virus infection	12	30.76923077	1.95E-10	IKBKB, CXCL10, IL6, JUN, MAPK8, CHUK, IRF3, IFNA1, IFNB1, MAPK14, MAP3K7, RIGI
hsa04657:IL-17 signaling pathway	9	23.07692308	2.50E-09	IKBKB, CXCL10, IL6, JUN, MAPK8, CXCL8, CHUK, MAPK14, MAP3K7
hsa05167:Kaposi sarcoma-associated herpesvirus infection	11	28.20512821	2.62E-09	IKBKB, IL6, JUN, NRAS, MAPK8, CXCL8, CHUK, IRF3, IFNA1, IFNB1, MAPK14
hsa05130:Pathogenic Escherichia coli infection	11	28.20512821	3.04E-09	PYCARD, IKBKB, IL6, JUN, MAPK8, CXCL8, CHUK, NCL, TNFSF10, MAPK14, MAP3K7
hsa04936:Alcoholic liver disease	10	25.64102564	3.06E-09	IKBKB, IL6, MAPK8, CXCL8, CHUK, IRF3, IFNA1, IFNB1, MAPK14, MAP3K7
hsa04625:C-type lectin receptor signaling pathway	9	23.07692308	5.64E-09	PYCARD, IKBKB, IL6, JUN, NRAS, MAPK8, CHUK, CD209, MAPK14
hsa05170:Human immunodeficiency virus 1 infection	10	25.64102564	1.02E-07	IKBKB, JUN, NRAS, MAPK8, CHUK, IRF3, IFNA1, IFNB1, MAPK14, MAP3K7
hsa05142:Chagas disease	8	20.51282051	1.26E-07	IKBKB, IL6, JUN, MAPK8, CXCL8, CHUK, IFNB1, MAPK14
hsa05131:Shigellosis	10	25.64102564	3.75E-07	PYCARD, IKBKB, JUN, MAPK8, CXCL8, CHUK, IRF3, IFNB1, MAPK14, MAP3K7

hsa05132:Salmonella infection	10	25.64102564	4.02E-07	PYCARD, IKBKB, IL6, JUN, MAPK8, CXCL8, CHUK, TNFSF10, MAPK14, MAP3K7
hsa05133:Pertussis	7	17.94871795	4.83E-07	PYCARD, IL6, JUN, MAPK8, CXCL8, IRF3, MAPK14
hsa05418:Fluid shear stress and atherosclerosis	8	20.51282051	1.05E-06	IKBKB, JUN, MAPK8, CHUK, SUMO1, MAPK14, MAP3K7, NFE2L2
hsa05208:Chemical carcinogenesis - reactive oxygen species	9	23.07692308	2.11E-06	IKBKB, NDUFA13, JUN, NRAS, MAPK8, CHUK, MAPK14, SOD1, NFE2L2
hsa04932:Non-alcoholic fatty liver disease	8	20.51282051	2.19E-06	IKBKB, IL6, NDUFA13, ITCH, JUN, MAPK8, CXCL8, MAPK14
hsa05163:Human cytomegalovirus infection	9	23.07692308	2.25E-06	IKBKB, IL6, NRAS, CXCL8, CHUK, IRF3, IFNA1, IFNB1, MAPK14
hsa05160:Hepatitis C	8	20.51282051	2.39E-06	IKBKB, CXCL10, NRAS, CHUK, IRF3, IFNA1, IFNB1, RIGI
hsa04933:AGE-RAGE signaling pathway in diabetic complications	7	17.94871795	2.46E-06	IL6, JUN, NRAS, MAPK8, CXCL8, FN1, MAPK14
hsa04660:T cell receptor signaling pathway	7	17.94871795	3.10E-06	IKBKB, JUN, NRAS, MAPK8, CHUK, MAPK14, MAP3K7
hsa04659:Th17 cell differentiation	7	17.94871795	3.86E-06	IKBKB, IL6, JUN, MAPK8, CHUK, MAPK14, RUNX1
hsa05120:Epithelial cell signaling in Helicobacter pylori infection	6	15.38461538	7.90E-06	IKBKB, JUN, MAPK8, CXCL8, CHUK, MAPK14
hsa04380:Osteoclast differentiation	7	17.94871795	1.03E-05	IKBKB, JUN, MAPK8, CHUK, IFNB1, MAPK14, MAP3K7
hsa04068:FoxO signaling pathway	7	17.94871795	1.18E-05	IKBKB, IL6, NRAS, MAPK8, CHUK, TNFSF10, MAPK14
hsa04210:Apoptosis	7	17.94871795	1.46E-05	IKBKB, JUN, NRAS, MAPK8, CHUK, CASP10, TNFSF10
hsa05200:Pathways in cancer	11	28.20512821	2.94E-05	IKBKB, IL6, JUN, NRAS, MAPK8, CXCL8, CHUK, IFNA1, FN1, NFE2L2, RUNX1
hsa05152:Tuberculosis	7	17.94871795	7.13E-05	IL6, MAPK8, IFNA1, IFNB1, CD209, CASP10, MAPK14
hsa05165:Human papillomavirus infection	8	20.51282051	2.90E-04	IKBKB, NRAS, CHUK, IRF3, IFNA1, IFNB1, FN1, SCRIB
hsa05235:PD-L1 expression and PD-1 checkpoint pathway in cancer	5	12.82051282	4.24E-04	IKBKB, JUN, NRAS, CHUK, MAPK14
hsa04658:Th1 and Th2 cell differentiation	5	12.82051282	4.81E-04	IKBKB, JUN, MAPK8, CHUK, MAPK14
hsa05168:Herpes simplex virus 1 infection	9	23.07692308	6.22E-04	IKBKB, NXF1, IL6, CHUK, IRF3, IFNA1, IFNB1, MAP3K7, RIGI
hsa04064:NF-kappa B signaling pathway	5	12.82051282	7.65E-04	IKBKB, CXCL8, CHUK, MAP3K7, RIGI
hsa04010:MAPK signaling pathway	7	17.94871795	0.001009226	IKBKB, JUN, NRAS, MAPK8, CHUK, MAPK14, MAP3K7
hsa05145:Toxoplasmosis	5	12.82051282	0.001010668	IKBKB, MAPK8, CHUK, MAPK14, MAP3K7
hsa04722:Neurotrophin signaling pathway	5	12.82051282	0.001266741	IKBKB, JUN, NRAS, MAPK8, MAPK14
hsa05166:Human T-cell leukemia virus 1 infection	6	15.38461538	0.001811466	IKBKB, IL6, JUN, NRAS, MAPK8, CHUK
hsa05221:Acute myeloid leukemia	4	10.25641026	0.002417346	IKBKB, NRAS, CHUK, RUNX1

hsa04151:PI3K-Akt signaling pathway	7	17.94871795	0.002610658	IKBKB, IL6, NRAS, CHUK, IFNA1, IFNB1, FN1
hsa05220:Chronic myeloid leukemia	4	10.25641026	0.003460909	IKBKB, NRAS, CHUK, RUNX1
hsa04217:Necroptosis	5	12.82051282	0.00365088	PYCARD, MAPK8, IFNA1, IFNB1, TNFSF10
hsa04662:B cell receptor signaling pathway	4	10.25641026	0.004288735	IKBKB, JUN, NRAS, CHUK
hsa04912:GnRH signaling pathway	4	10.25641026	0.006097725	JUN, NRAS, MAPK8, MAPK14
hsa04060:Cytokine-cytokine receptor interaction	6	15.38461538	0.006154223	CXCL10, IL6, CXCL8, IFNA1, IFNB1, TNFSF10
hsa01523:Antifolate resistance	3	7.692307692	0.006421935	IKBKB, IL6, CHUK
hsa01522:Endocrine resistance	4	10.25641026	0.007049885	JUN, NRAS, MAPK8, MAPK14
hsa04062:Chemokine signaling pathway	5	12.82051282	0.007116274	IKBKB, CXCL10, NRAS, CXCL8, CHUK
hsa04061:Viral protein interaction with cytokine and cytokine receptor	4	10.25641026	0.007454149	CXCL10, IL6, CXCL8, TNFSF10
hsa04650:Natural killer cell mediated cytotoxicity	4	10.25641026	0.013976392	NRAS, IFNA1, IFNB1, TNFSF10
hsa04926:Relaxin signaling pathway	4	10.25641026	0.014884799	JUN, NRAS, MAPK8, MAPK14
hsa05010:Alzheimer disease	6	15.38461538	0.017951774	IKBKB, IL6, NDUFA13, NRAS, MAPK8, CHUK
hsa05134:Legionellosis	3	7.692307692	0.022017268	PYCARD, IL6, CXCL8
hsa05020:Prion disease	5	12.82051282	0.023351471	IL6, NDUFA13, MAPK8, MAPK14, SOD1
hsa04218:Cellular senescence	4	10.25641026	0.024557918	IL6, NRAS, CXCL8, MAPK14
hsa04530:Tight junction	4	10.25641026	0.030185023	JUN, MAPK8, SCRIB, RUNX1
hsa04664:Fc epsilon RI signaling pathway	3	7.692307692	0.030573338	NRAS, MAPK8, MAPK14
hsa04920:Adipocytokine signaling pathway	3	7.692307692	0.031407485	IKBKB, MAPK8, CHUK
hsa04917:Prolactin signaling pathway	3	7.692307692	0.032250658	NRAS, MAPK8, MAPK14
hsa04137:Mitophagy - animal	3	7.692307692	0.033963754	JUN, NRAS, MAPK8
hsa05212:Pancreatic cancer	3	7.692307692	0.037494687	IKBKB, MAPK8, CHUK
hsa05140:Leishmaniasis	3	7.692307692	0.038398776	JUN, MAPK14, MAP3K7
hsa05022:Pathways of neurodegeneration - multiple diseases	6	15.38461538	0.040550345	IL6, NDUFA13, NRAS, MAPK8, MAPK14, SOD1
hsa04012:ErbB signaling pathway	3	7.692307692	0.045926839	JUN, NRAS, MAPK8
hsa05210:Colorectal cancer	3	7.692307692	0.04690362	JUN, NRAS, MAPK8
hsa05203:Viral carcinogenesis	4	10.25641026	0.048438384	JUN, NRAS, IRF3, SCRIB
hsa05205:Proteoglycans in cancer	4	10.25641026	0.049025113	NRAS, FN1, MIR21, MAPK14

Supplementary Table S4: Gene ontology for Human H5N1 infection associated genes

A) **Biological Process**

GO terms	Count	%	P-value	Genes
GO:0098586~cellular response to virus	8	20.51282051	3.06E-10	CXCL10, IL6, CHUK, IRF3, IFNA1, IFNB1, MIR21, MAPK14
GO:0043065~positive regulation of apoptotic process	9	23.07692308	2.00E-07	PYCARD, IL6, JUN, MAPK8, UBD, TNFSF10, MIR21, SCRIB, SOD1
GO:0051607~defense response to virus	8	20.51282051	3.13E-07	PYCARD, IFITM3, IL6, ITCH, IRF3, IFNA1, IFNB1, RIGI
GO:0006954~inflammatory response	9	23.07692308	1.12E-06	PYCARD, CRP, IKBKB, CXCL10, IL6, ITCH, CXCL8, CHUK, NFE2L2
GO:0071276~cellular response to cadmium ion	5	12.82051282	1.23E-06	JUN, MAPK8, CHUK, SUMO1, SOD1
GO:0043123~positive regulation of I-kappaB kinase/NF-kappaB signaling	7	17.94871795	1.89E-06	IKBKB, CHUK, IRF3, CASP10, UBD, TNFSF10, MAP3K7
GO:0071222~cellular response to lipopolysaccharide	7	17.94871795	1.95E-06	PYCARD, CXCL10, IL6, MAPK8, CXCL8, MIR21, MAPK14
GO:0010628~positive regulation of gene expression	9	23.07692308	5.95E-06	CRP, IL6, MAPK8, CXCL8, FN1, MIR21, MAPK14, RIGI, NFE2L2
GO:0071356~cellular response to tumor necrosis factor	6	15.38461538	9.48E-06	PYCARD, IKBKB, CXCL8, CHUK, MAPK14, NFE2L2
GO:0045944~positive regulation of transcription from RNA polymerase II promoter	12	30.76923077	1.33E-05	IKBKB, CXCL10, IL6, JUN, CHUK, IRF3, IFNB1, NCL, MAPK14, RIGI, NFE2L2, RUNX1
GO:0009615~response to virus	5	12.82051282	6.57E-05	IFITM3, IKBKB, CHUK, IFNB1, RIGI
GO:0060337~type I interferon signaling pathway	4	10.25641026	9.43E-05	IFITM3, IRF3, IFNA1, IFNB1
GO:0001525~angiogenesis	6	15.38461538	1.28E-04	CEACAM1, JUN, CXCL8, NCL, FN1, MAPK14
GO:0045087~innate immune response	8	20.51282051	1.46E-04	PYCARD, CRP, IKBKB, ITCH, CHUK, CD209, SFTPD, RIGI
GO:0006915~apoptotic process	8	20.51282051	1.58E-04	PYCARD, ITCH, JUN, IRF3, CASP10, TNFSF10, MAPK14, SOD1
GO:0007252~I-kappaB phosphorylation	3	7.692307692	2.46E-04	IKBKB, CHUK, MAP3K7
GO:0051403~stress-activated MAPK cascade	3	7.692307692	5.03E-04	IKBKB, MAPK8, MAP3K7
GO:0071360~cellular response to exogenous dsRNA	3	7.692307692	6.31E-04	IRF3, IFNB1, RIGI
GO:0032088~negative regulation of NF-kappaB transcription factor activity	4	10.25641026	7.83E-04	PYCARD, ITCH, CHUK, MIR21
GO:0038095~Fc-epsilon receptor signaling pathway	3	7.692307692	9.30E-04	IKBKB, MAPK8, MAP3K7
GO:0032727~positive regulation of interferon-alpha production	3	7.692307692	0.001189786	CHUK, IRF3, RIGI
GO:0043330~response to exogenous dsRNA	3	7.692307692	0.002033482	IFNA1, IFNB1, RIGI
GO:0072593~reactive oxygen species metabolic process	3	7.692307692	0.002277938	NDUFA13, SFTPD, SOD1
GO:0034614~cellular response to reactive oxygen species	3	7.692307692	0.002535599	JUN, MAPK8, CHUK

GO:0006955~immune response	6	15.38461538	0.002639876	IFITM3, FYB1, CXCL8, CHUK, TNFSF10, MARCHF8
GO:0006953~acute-phase response	3	7.692307692	0.003090132	CRP, IL6, FN1
GO:0045071~negative regulation of viral genome replication	3	7.692307692	0.003696272	IFITM3, IFNB1, MIR141
GO:0051092~positive regulation of NF-kappaB transcription factor activity	4	10.25641026	0.003808581	PYCARD, IKBKB, CHUK, MAP3K7
GO:0045766~positive regulation of angiogenesis	4	10.25641026	0.004081234	CXCL8, MIR21, NFE2L2, RUNX1
GO:0050766~positive regulation of phagocytosis	3	7.692307692	0.004353218	PYCARD, SFTPD, SOD1
GO:0043280~positive regulation of cysteine-type endopeptidase activity involved in apoptotic process	3	7.692307692	0.005060177	PYCARD, NDUFA13, TNFSF10
GO:0033209~tumor necrosis factor-mediated signaling pathway	3	7.692307692	0.005244639	PYCARD, IKBKB, CHUK
GO:0018105~peptidyl-serine phosphorylation	4	10.25641026	0.005371472	IKBKB, MAPK8, CHUK, MAPK14
GO:0031281~positive regulation of cyclase activity	2	5.128205128	0.00589298	MAPK8, MAPK14
GO:0006959~humoral immune response	3	7.692307692	0.006415349	IL6, IFNA1, IFNB1
GO:0007249~I-kappaB kinase/NF-kappaB signaling	3	7.692307692	0.006415349	IKBKB, CHUK, MAP3K7
GO:0032757~positive regulation of interleukin-8 production	3	7.692307692	0.007255831	PYCARD, IL6, RIGI
GO:0000187~activation of MAPK activity	3	7.692307692	0.009077402	MAPK14, MAP3K7, SOD1
GO:0070423~nucleotide-binding oligomerization domain containing signaling pathway	2	5.128205128	0.009802834	ITCH, MAP3K7
GO:0045893~positive regulation of transcription, DNA-templated	6	15.38461538	0.013250311	IKBKB, IL6, JUN, CHUK, NUP98, RUNX1
GO:2000660~negative regulation of interleukin-1-mediated signaling pathway	2	5.128205128	0.013697714	IL6, MIR21
GO:0030853~negative regulation of granulocyte differentiation	2	5.128205128	0.015639555	CEACAM1, RUNX1
GO:0007179~transforming growth factor beta receptor signaling pathway	3	7.692307692	0.016852104	JUN, MIR21, MAP3K7
GO:0032755~positive regulation of interleukin-6 production	3	7.692307692	0.017486489	PYCARD, IL6, RIGI
GO:0032760~positive regulation of tumor necrosis factor production	3	7.692307692	0.019116353	PYCARD, IL6, RIGI
GO:0030334~regulation of cell migration	3	7.692307692	0.019449768	CEACAM1, MIR21, RIGI
GO:0010888~negative regulation of lipid storage	2	5.128205128	0.019512078	CRP, IL6

GO:0007229~integrin-mediated signaling pathway	3	7.692307692	0.019785639	CEACAM1, FYB1, FN1
GO:0050852~T cell receptor signaling pathway	3	7.692307692	0.020464708	IKBKB, FYB1, MAP3K7
GO:0042058~regulation of epidermal growth factor receptor signaling pathway	2	5.128205128	0.021442772	CEACAM1, MIR21
GO:0008284~positive regulation of cell proliferation	5	12.82051282	0.022365613	CXCL10, IL6, MIR141, FN1, MIR21
GO:0051091~positive regulation of sequence-specific DNA binding transcription factor activity	3	7.692307692	0.022917299	PYCARD, IL6, RIGI
GO:0006935~chemotaxis	3	7.692307692	0.024370778	CXCL10, CXCL8, MAPK14
GO:0038066~p38MAPK cascade	2	5.128205128	0.025293062	MAPK14, MAP3K7
GO:0050830~defense response to Gram-positive bacterium	3	7.692307692	0.026620189	PYCARD, CRP, IL6
GO:1904996~positive regulation of leukocyte adhesion to vascular endothelial cell	2	5.128205128	0.027212671	IL6, MIR21
GO:0010952~positive regulation of peptidase activity	2	5.128205128	0.0291286	NDUFA13, FN1
GO:0010803~regulation of tumor necrosis factor-mediated signaling pathway	2	5.128205128	0.031040853	PYCARD, IKBKB
GO:0051146~striated muscle cell differentiation	2	5.128205128	0.031040853	CHUK, MAPK14
GO:0030194~positive regulation of blood coagulation	2	5.128205128	0.03294944	ST3GAL4, NFE2L2
GO:0032480~negative regulation of type I interferon production	2	5.128205128	0.03294944	ITCH, MIR21
GO:0007165~signal transduction	7	17.94871795	0.03586512	PYCARD, CXCL10, CEACAM1, NRAS, CXCL8, TNFSF10, MAPK14
GO:0002323~natural killer cell activation involved in immune response	2	5.128205128	0.036755637	IFNA1, IFNB1
GO:2000406~positive regulation of T cell migration	2	5.128205128	0.036755637	PYCARD, CXCL10
GO:0060088~auditory receptor cell stereocilium organization	2	5.128205128	0.036755637	SCRIB, SOD1
GO:0019221~cytokine-mediated signaling pathway	3	7.692307692	0.037716332	IL6, IFNA1, IFNB1
GO:0035994~response to muscle stretch	2	5.128205128	0.038653262	JUN, MAPK14
GO:0032930~positive regulation of superoxide anion generation	2	5.128205128	0.040547247	CRP, SOD1
GO:0033141~positive regulation of peptidyl-serine phosphorylation of STAT protein	2	5.128205128	0.040547247	IFNA1, IFNB1
GO:0070498~interleukin-1-mediated signaling pathway	2	5.128205128	0.040547247	IKBKB, MAP3K7
GO:0002223~stimulatory C-type lectin receptor signaling pathway	2	5.128205128	0.042437598	IKBKB, MAP3K7

GO:0035458~cellular response to interferon-beta	2	5.128205128	0.046207428	NDUFA13, IFNB1
GO:0048026~positive regulation of mRNA splicing, via spliceosome	2	5.128205128	0.049962805	NCL, NUP98
GO:1904753~negative regulation of vascular associated smooth muscle cell migration	2	5.128205128	0.049962805	MIR21, NFE2L2
GO:0034341~response to interferon-gamma	2	5.128205128	0.049962805	IFITM3, UBD
GO:0002286~T cell activation involved in immune response	2	5.128205128	0.049962805	IFNA1, IFNB1

B) Cellular Components

GO terms	Count	%	P-value	Genes
GO:0005576~extracellular region	14	35.8974359	6.79E-05	CRP, CXCL8, IFNA1, IFNB1, SFTPD, FN1, MAPK14, SOD1, PYCARD, CXCL10, IL6, CD209, TNFSF10, ST3GAL4
GO:0008385~IkappaB kinase complex	3	7.692307692	6.94E-05	PYCARD, IKBKB, CHUK
GO:0005615~extracellular space	12	30.76923077	5.53E-04	CRP, CXCL10, IL6, CXCL8, IFNA1, IFNB1, MIR141, SFTPD, TNFSF10, FN1, MIR21, SOD1
GO:0005654~nucleoplasm	17	43.58974359	0.00102122	NDUFA13, JUN, CHUK, SCRIB, MAPK14, SOD1, RUNX1, PYCARD, NXF1, ITCH, MAPK8, IRF3, SUMO1, UBD, NCL, NUP98, NFE2L2
GO:0005829~cytosol	19	48.71794872	0.005210377	JUN, FYB1, CHUK, MAPK14, SOD1, PYCARD, IKBKB, NXF1, ITCH, NRAS, MAPK8, IRF3, SUMO1, CASP10, UBD, NUP98, MAP3K7, RIGI, NFE2L2
GO:0005643~nuclear pore	3	7.692307692	0.012799343	NXF1, SUMO1, NUP98
GO:0005737~cytoplasm	18	46.15384615	0.014630039	NDUFA13, CHUK, MIR21, MAPK14, SOD1, PYCARD, IKBKB, NXF1, ITCH, MAPK8, IRF3, CASP10, CD209, UBD, NCL, MARCHF8, RIGI, NFE2L2
GO:0035631~CD40 receptor complex	2	5.128205128	0.020146809	IKBKB, CHUK
GO:0042405~nuclear inclusion body	2	5.128205128	0.023767158	NXF1, NUP98
GO:0032991~macromolecular complex	5	12.82051282	0.039911291	PYCARD, IFITM3, ITCH, FYB1, SOD1
GO:1990904~ribonucleoprotein complex	3	7.692307692	0.044925141	NCL, NUP98, RIGI
GO:0031901~early endosome membrane	3	7.692307692	0.046255319	IFITM3, ITCH, MARCHF8
GO:0070062~extracellular exosome	9	23.07692308	0.046656831	ITCH, CEACAM1, NRAS, NCL, TNFSF10, FN1, MIR21, SCRIB, SOD1

C) **Molecular functions**

GO terms	Count	%	P-value	Genes
GO:0042802~identical protein binding	13	33.33333333	8.73E-05	CRP, JUN, SFTPD, FN1, SOD1, PYCARD, IKBKB, CEACAM1, IRF3, NCL, TNFSF10, MAP3K7, RIGI
GO:0005515~protein binding	36	92.30769231	2.37E-04	IFITM3, CRP, NDUFA13, CXCL8, IFNA1, IKBKB, PYCARD, NXF1, NRAS, MAPK8, SUMO1, CASP10, UBD, TNFSF10, MARCHF8, MAP3K7, JUN, FYB1, CHUK, IFNB1, SFTPD, FN1, SCRIB, MAPK14, RUNX1, SOD1, CXCL10, ITCH, IL6, CEACAM1, IRF3, CD209, NCL, NUP98, RIGI, NFE2L2
GO:0045236~CXCR chemokine receptor binding	3	7.692307692	4.08E-04	CXCL10, ITCH, CXCL8
GO:0004707~MAP kinase activity	3	7.692307692	4.66E-04	MAPK8, MAPK14, MAP3K7
GO:0019899~enzyme binding	6	15.38461538	0.001052677	PYCARD, JUN, MAPK8, SUMO1, FN1, MAPK14
GO:0044389~ubiquitin-like protein ligase binding	3	7.692307692	0.001059921	ITCH, JUN, SUMO1
GO:0008384~IkappaB kinase activity	2	5.128205128	0.006029816	IKBKB, CHUK
GO:0005125~cytokine activity	4	10.25641026	0.006734932	IL6, IFNA1, IFNB1, TNFSF10
GO:0004674~protein serine/threonine kinase activity	5	12.82051282	0.007754702	IKBKB, MAPK8, CHUK, MAPK14, MAP3K7
GO:0097110~scaffold protein binding	3	7.692307692	0.008510377	IKBKB, CHUK, MAP3K7
GO:0000976~transcription regulatory region sequence-specific DNA binding	4	10.25641026	0.012214656	JUN, IRF3, NFE2L2, RUNX1
GO:0042803~protein homodimerization activity	6	15.38461538	0.013813531	PYCARD, IKBKB, CEACAM1, CHUK, IRF3, RUNX1
GO:0005138~interleukin-6 receptor binding	2	5.128205128	0.014014526	PYCARD, IL6
GO:0019903~protein phosphatase binding	3	7.692307692	0.015056416	CEACAM1, MAPK8, MAPK14
GO:0097153~cysteine-type endopeptidase activity involved in apoptotic process	2	5.128205128	0.019962039	PYCARD, CASP10
GO:1990459~transferrin receptor binding	2	5.128205128	0.021936769	IKBKB, CHUK
GO:0031625~ubiquitin protein ligase binding	4	10.25641026	0.023450741	JUN, SUMO1, CASP10, RIGI
GO:0005524~ATP binding	8	20.51282051	0.032522772	IKBKB, NDUFA13, MAPK8, CHUK, MAPK14, MAP3K7, RIGI, RUNX1
GO:0005132~type I interferon receptor binding	2	5.128205128	0.033704047	IFNA1, IFNB1
GO:0004672~protein kinase activity	4	10.25641026	0.040407787	IKBKB, CHUK, MAPK14, MAP3K7
GO:0005102~receptor binding	4	10.25641026	0.045335642	CXCL10, FYB1, TNFSF10, FN1
GO:0008201~heparin binding	3	7.692307692	0.048850691	CXCL10, CXCL8, FN1

Supplementary Table S5: Top ten hub genes by their rank based on MCC analysis

Rank	MCC	Degree	MNC	DMNC
1	IL6	JUN	JUN	TNFSF10
2	CXCL8	IL6	IL6	PYCARD
3	JUN	IKBKB	IKBKB	CXCL10
4	IKBKB	CXCL8	CXCL8	CHUK
5	DDX58	DDX58	DDX58	CD209
6	CXCL10	IFNB1	IFNB1	IFNA1
7	IFNB1	IFNA1	IFNA1	IRF3
8	IFNA1	CXCL10	CXCL10	IFNB1
9	IRF3	IRF3	IRF3	CRP
10	CHUK	CHUK	CHUK	CXCL8

Supplementary Figure S1: Protein-protein interactions for Local Topological Analysis Methods

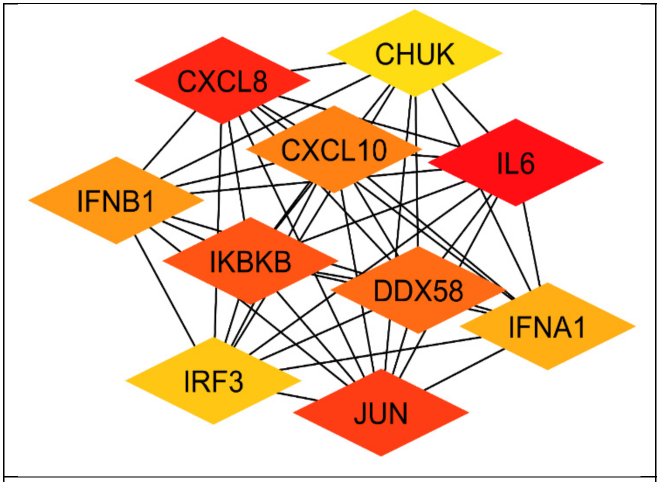

A. MCC

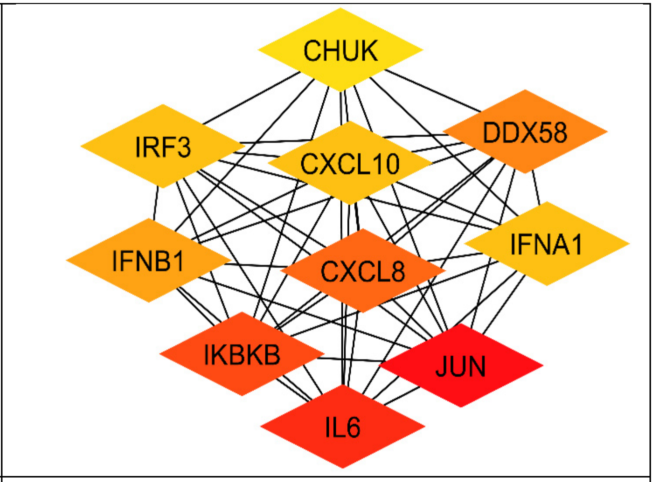

B. Degree

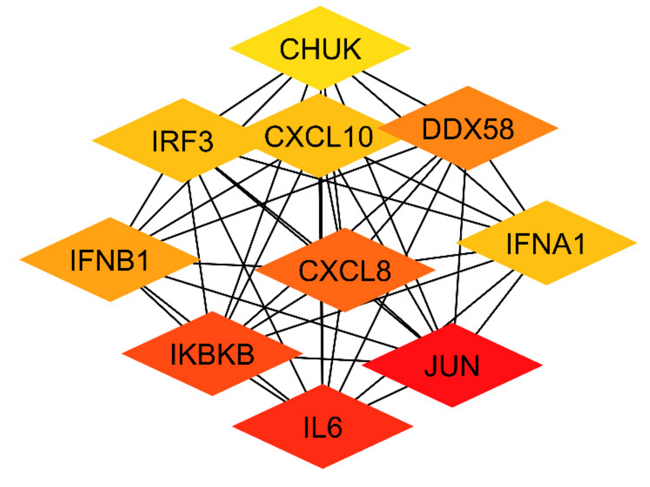

C. MNC

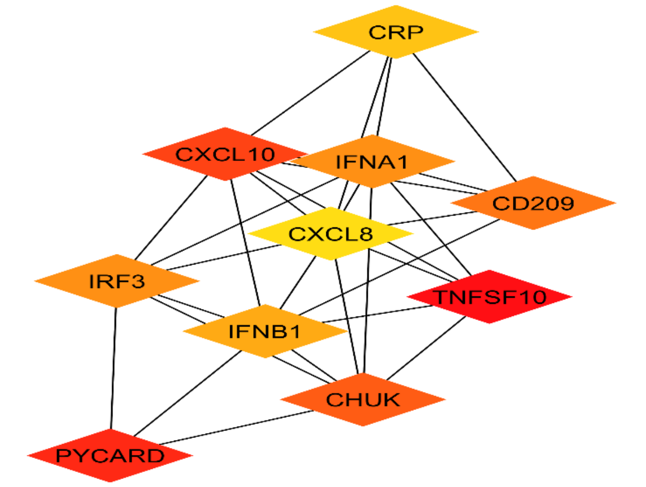

D. DMNC
